# Self-Reported Symptom Burden and Clinical Characteristics in Fibromyalgia: Evidence from a Large Online Survey in Italy

**DOI:** 10.3390/medicina62071319

**Published:** 2026-07-08

**Authors:** Michael Tenti, Barbara Suzzi, Catia Bugli, Sabrina Travaglini Albanesi, Elisa Lombardi, Lucia Lovecchio, Camilla Ghedini, William Raffaeli

**Affiliations:** 1 ISAL Foundation—ETS, Institute for Research on Pain, 47921 Rimini, Italy; 2 Comitato Fibromialgici Uniti (CFU)—Italia Odv, 40055 Castenaso, Italy; presidenza@cfuitalia.it (B.S.);

**Keywords:** fibromyalgia, chronic pain, self-report, online survey, sampling bias, measurement bias, selection bias, patient-reported outcome measures, Fibromyalgia Impact Questionnaire—Revised, Widespread Pain Index

## Abstract

*Background and Objectives*: Fibromyalgia (FM) is a chronic primary pain syndrome. Its hallmark symptom is widespread pain, often accompanied by fatigue, sleep disturbances, and cognitive symptoms. Online surveys efficiently collect patient-reported data, but individuals recruited through this approach remain poorly characterized. This study aimed to describe a large online cohort of individuals with FM, providing data for comparison with the Italian Fibromyalgia Registry (IFR). *Materials and Methods*: Participants who self-reported a physician diagnosis of FM completed an online survey assessing socio-demographic and clinical characteristics, treatments and their perceived effectiveness, lifestyle, and impact variables. Disease severity and symptom burden were assessed using the Fibromyalgia Impact Questionnaire—Revised (FIQR), Widespread Pain Index (WPI), and Symptom Severity Scale (SSS). Participants were classified according to the 2016 American College of Rheumatology (ACR) criteria. Descriptive statistics were computed, and exploratory analyses assessed sex differences and differences according to ACR status using independent-samples *t*-tests with Bonferroni correction. *Results*: A total of 6022 participants were included (mean age 52.3 ± 10.3 years, 96.7% female). Nearly half (49.6%) reported pain duration > 10 years, and 50.4% had received conflicting diagnoses. Disease burden was high, with >70% classified as moderate-to-severe according to the FIQR. Overall, 79.4% fulfilled the 2016 ACR criteria. Participants not fulfilling the criteria showed lower symptom severity, although considerable clinical overlap was observed between groups. Pharmacological treatments were used by 81.1% of participants and non-pharmacological approaches by 55.1%, both with moderate perceived effectiveness. FM substantially affected daily life, particularly work and social functioning. After correction for multiple comparisons, sex differences were limited to FIQR functioning and symptom domains, with small effect sizes. *Conclusions*: Compared with published registry-based data, participants recruited through this online survey reported a higher symptom burden and longer pain duration, while showing a broadly similar symptom profile. Although the descriptive nature of the comparison precludes causal inferences, the findings suggest that online surveys and clinical registries may provide complementary perspectives on the FM population.

## 1. Introduction

Chronic pain, i.e., pain that persists or recurs for more than three months, is widely recognized as a complex and multifactorial condition [1]. In Italy, chronic pain affects 24.1% of the adult population and is more prevalent among women [2], consistent with international epidemiological data [3]. Chronic pain encompasses a broad spectrum of conditions, including, among others, migraine, low back pain, osteoarthritis, and neuropathic pain syndromes, reflecting its heterogeneous clinical nature. Recently, chronic pain has been systematized in the ICD-11, which distinguishes between chronic primary pain and chronic secondary pain. The former refers to persistent or recurrent pain associated with emotional distress or functional disability, not better explained by another condition, whereas the latter arises as a symptom of an underlying disease [4].

Among chronic primary pain conditions, fibromyalgia (FM) represents one of the most challenging syndromes, both for patients, in terms of adaptation to its persistent and multifaceted symptoms, and for healthcare professionals, with regard to its diagnosis and management [5,6]. FM is a chronic primary pain condition whose hallmark symptom is widespread pain, often accompanied by fatigue, sleep disturbances, cognitive difficulties, and a range of somatic and psychological symptoms that significantly impair quality of life [4,7]. It has been recognized as a distinct clinical entity since the Copenhagen Declaration of 1992, which formally acknowledged FM as a specific disease condition [8]. Despite its high prevalence and substantial burden at both individual and societal levels [9,10,11], FM remains a complex and frequently underrecognized condition. Persistent challenges in diagnosis, management, and epidemiological characterization continue to hinder its clinical management [6,12], also due to the lack of validated diagnostic tests and reliable biomarkers [13].

Epidemiological studies have consistently shown that FM disproportionately affects women, with prevalence estimates ranging between 2% and 4% in the general population across different countries. However, these estimates vary widely depending on diagnostic criteria, study design, and sampling methods [14,15,16]. This variability reflects, at least in part, the intrinsic complexity of FM, which is characterized by diagnostic ambiguity, heterogeneous clinical presentations, and challenges in case identification [17,18]. Indeed, the time to diagnosis remains considerable, and a substantial proportion of individuals remain undiagnosed or untreated, further complicating efforts to accurately define the epidemiology and clinical features of the condition [19,20,21].

In this context, improving the characterization of FM requires the systematic collection of large-scale real-world data capturing the full spectrum of patient experiences across different clinical settings. Patient registries represent a valuable tool in this regard, as they allow for the standardized collection of longitudinal clinical and socio-demographic data in well-defined populations [22]. In Italy, the establishment of the Italian Fibromyalgia Registry (IFR) in 2019 marked a significant step forward, providing a structured, web-based platform for the collection of real-world data from patients with FM across multiple centers. The registry aims to monitor disease trajectories, support clinical decision-making, and facilitate research initiatives [23].

Research based on registry data offers important advantages, including access to large, well-characterized populations and the possibility of examining clinical and socio-demographic variables over time. However, these approaches have important limitations. In particular, patients included in clinical registries are often those who have accessed specialized care, potentially excluding individuals with limited access to healthcare services or those who remain undiagnosed or untreated. Moreover, registries are not primarily designed to address specific research questions, and the available data may be incomplete or lack key variables of interest. As observational data sources, they are also vulnerable to different forms of bias, including selection bias, information bias, and confounding [24,25,26]. Consequently, registry-based samples may not fully reflect the heterogeneity of the FM population.

In recent years, online surveys have become increasingly common in health research [27], including for the investigation of chronic conditions such as FM [28,29,30,31,32,33,34,35,36,37]. Digital recruitment strategies enable researchers to rapidly reach large and geographically diverse populations at relatively low cost, including individuals who may be difficult to access through traditional research settings, such as those not engaged with healthcare services or members of stigmatized groups. This approach is particularly relevant for conditions like FM, where delays in diagnosis, stigma, and variability in healthcare pathways may influence whether individuals engage with formal healthcare systems [6,38]. However, online surveys also present important methodological challenges. Sampling procedures are often less controlled, and it is frequently difficult to define a clear sampling frame or verify participants’ characteristics, as data are typically self-reported. Self-selection bias represents a major limitation, as individuals who choose to participate may differ systematically from those who do not, potentially leading to the overrepresentation of certain subgroups, such as those with higher symptom burden or greater engagement in online communities. Additional concerns include the possibility of multiple responses, inaccuracies in reported information, and difficulties in estimating response rates. More broadly, these issues may limit the generalizability of findings and the ability to accurately represent the target population [39,40].

Against this background, data derived from online surveys may provide a complementary perspective to that offered by established clinical registries, potentially capturing individuals who are underrepresented in traditional clinical settings. In this context, the present study aimed to describe the socio-demographic and clinical characteristics of a large sample of individuals with FM recruited through an online survey. Particular attention was paid to key variables such as age, sex distribution, educational level, employment status, geographic distribution, and FM-related symptoms. By providing a detailed characterization of this sample, the study seeks to contribute to a more comprehensive understanding of the heterogeneity of the FM population and to explore how individuals recruited through online surveys compare with those represented in registry-based studies. A better understanding of the characteristics associated with different recruitment strategies may help inform future research designs and support the integration of complementary data sources in FM research.

The diagnosis of FM is currently based on the 2016 revised American College of Rheumatology (ACR) criteria, which integrate patient-reported measures of widespread pain and symptom severity [41]. These criteria rely on the Widespread Pain Index (WPI) and the Symptom Severity Scale (SSS) and require the presence of generalized pain across multiple body regions together with a minimum level of symptom severity. The use of these standardized instruments is recommended in research settings to ensure consistent case definition and comparability across studies. In this context, a secondary aim of the study was to assess whether participants reporting a previous diagnosis of FM also fulfilled the 2016 ACR criteria.

## 2. Materials and Methods

### 2.1. Study Design and Participants

A cross-sectional survey was conducted through a questionnaire that was developed through a participatory process and administered online to a convenience sample of individuals reporting a diagnosis of FM. The study was carried out between May 2025 and October 2025.

Participants were eligible if they were 18 years or older and self-reported having received a physician diagnosis of FM. No independent verification of the diagnosis was performed. Participation was voluntary and anonymous.

### 2.2. Recruitment Procedures

Participants were recruited through multiple online channels associated with the Italian FM patient association CFU-Italia ODV. Specifically, the survey link was disseminated via posts and articles published on CFU-Italia ODV’s Facebook page and website.

In addition, the link was shared within the association’s internal communication networks, including WhatsApp broadcast lists and group chats managed by regional representatives (FM patients themselves), which are routinely used for communication with registered members. Further recruitment was supported by members of the scientific committee of CFU-Italia (healthcare professionals), who shared the survey link through their professional networks and communication channels.

The survey was also disseminated by partner organizations, including ISAL Foundation (Institute of Algological Sciences), an Italian non-profit organization dedicated to research, education, and patient support in the field of chronic pain.

### 2.3. Survey Development and Content

The online survey was developed through a participatory process involving the scientific committee of CFU-Italia, regional representatives of the association, and external experts from ISAL Foundation. The selection of variables and instruments was guided by the need to ensure clinical relevance, comparability with registry-based data (in particular the IFR), and minimization of respondent burden.

The questionnaire was administered online and consisted of several sections.

#### 2.3.1. Informed Consent

The first section included detailed information about the study and required participants to provide informed consent before accessing the questionnaire.

#### 2.3.2. Socio-Demographic and Clinical Variables

Participants were asked to report socio-demographic information, including age, sex, region of residence, living situation, educational level, and employment status. Clinical information included duration of pain (categorized as <1 year, 1–3 years, 4–5 years, 6–10 years, and >10 years), self-reported physician diagnosis of FM (yes/no), and the specialist who provided the diagnosis (rheumatologist, pain specialist, neurologist, orthopedist, other).

Participants were also asked to indicate the time lag between symptom onset and diagnosis (categorized as <1 year, 1–3 years, 4–5 years, 6–10 years, and >10 years) and whether they had ever received conflicting medical opinions regarding FM (yes/no).

#### 2.3.3. Fibromyalgia-Specific Measures

Fibromyalgia symptomatology was assessed using the Widespread Pain Index (WPI) and the Symptom Severity Scale (SSS), which together form the Polysymptomatic Distress Scale (PDS), a tool developed from the 2016 ACR FM criteria to measure disease severity [41]. The WPI assesses the presence (1) or absence (0) of pain in 19 specified non-articular body regions, typically indicated on a front and back body diagram. The total WPI score ranges from 0 to 19, with higher scores reflecting a greater extent of widespread pain. The SSS evaluates the severity of core non-pain symptoms, including fatigue, unrefreshing sleep, and cognitive difficulties, each rated on a scale from 0 (no problem) to 3 (severe problem). In addition, it includes the presence (score = 1) or absence (score = 0) of further symptoms. The total SSS score ranges from 0 to 12, with higher scores indicating greater symptom severity. The combined PDS score is obtained by summing the WPI and SSS scores, resulting in a total score ranging from 0 to 31, with higher scores indicating greater overall disease severity.

Disease impact was assessed using the Revised Fibromyalgia Impact Questionnaire (FIQR) [42], which consists of 21 numerical rating scales (range 0–10, with 10 indicating the worst condition). The FIQR evaluates three domains: function, overall impact, and symptoms, referring to the previous seven days. The total score ranges from 0 to 100, with higher scores indicating greater disease severity. For interpretive purposes, FIQR scores can be categorized into different levels of severity: scores between 0 and 30 are indicative of remission or minimal impact, 31–45 correspond to mild severity, 46–65 to moderate severity, and values above 65 reflect high disease severity [43].

#### 2.3.4. Treatment-Related Variables

Participants were asked whether they were currently taking medications for pain management (yes/no), and those answering yes were asked to rate perceived effectiveness on a 0–10 Likert scale. A similar set of questions was administered regarding the use of medical cannabis for pain control.

Non-pharmacological treatments were also assessed. Participants were asked whether they engaged in such treatments (yes/no) and, if so, to specify which among a predefined list (acupuncture, physiotherapy, psychotherapy, gentle exercise, phytotherapy, meditation, thermal treatments, tai chi, yoga, other); in this regard, participants could select more than one non-pharmacological treatment. Perceived effectiveness of these treatments was rated on a 0–10 Likert scale.

#### 2.3.5. Lifestyle and Impact Variables

Participants were asked whether they had modified their diet following the FM diagnosis (yes/no). In addition, the perceived impact of FM on different domains of daily life was assessed using numerical rating scales (0–10), including family life, sexual life, work, and social functioning.

### 2.4. Operationalization of the 2016 ACR Criteria

FM classification was based on the 2016 ACR criteria. These criteria were operationalized in three components using self-reported questionnaire data. First, the WPI was used to quantify the number of painful body regions (0–19), while the SSS was used to assess the severity of associated symptoms (0–12). Second, the generalized pain criterion was operationalized according to the 2016 ACR definition [41]. Participants were classified as having generalized pain when they reported pain in at least four of five body regions: (1) left upper region, (2) right upper region, (3) left lower region, (4) right lower region, and (5) axial region. These regions were derived from the individual pain locations indicated in the WPI checklist. Third, symptom duration of at least three months, as required by the ACR criteria, was assessed through a specific item administered at the end of the SSS, in which participants were asked to report whether their symptoms had been present at a similar level for a minimum duration of three months.

Participants were classified as fulfilling the 2016 ACR criteria when they met all of the following conditions: (i) presence of generalized pain (≥4 of 5 body regions), (ii) symptom duration of at least three months, and (iii) fulfillment of WPI/SSS threshold combinations (WPI ≥ 7 and SSS ≥ 5, or WPI 4–6 and SSS ≥ 9).

### 2.5. Ethical Considerations

Formal Institutional Review Board approval was not required for this study under applicable national and European regulations applicable to non-interventional, fully anonymous survey-based research (Regulation EU 536/2014; Regulation EU 2017/745; D. Lgs. 52/2019) [44,45,46]. Ethics committee approval is generally required for interventional clinical trials, studies involving investigational medicinal products or medical devices, observational studies using identifiable clinical data, biological samples, studies involving biological samples, non-anonymized clinical data from patient registries, or other sensitive data.

Under the General Data Protection Regulation (GDPR) (EU 2016/679) [47], personal data are defined as any information relating to an identified or identifiable natural person, while special categories of personal data (often referred to as sensitive data) receive additional protection. However, such provisions apply only when data are not fully anonymous. According to Recital 26 of the GDPR, the principles of data protection do not apply to anonymous information, i.e., data that do not relate to an identified or identifiable natural person.

The present study was a non-interventional, cross-sectional online survey conducted in the general population of individuals with fibromyalgia. No clinical intervention, biological sampling, or access to identifiable clinical records was involved. Data were collected anonymously via Google Forms, which was designed according to a privacy-by-design approach to prevent the collection of any directly or indirectly identifiable personal information (including names, contact details, precise dates of birth, email addresses, or IP addresses). All procedures were conducted in accordance with the principles of the Declaration of Helsinki [48]. Prior to participation, all respondents were presented with information describing the study objectives, the voluntary nature of participation, and data management procedures. In line with the information provided, informed consent was considered granted upon electronic initiation and completion of the survey.

### 2.6. Data Analysis

All statistical analyses were conducted using IBM SPSS Statistics version 25.0 (IBM Corp., Armonk, NY, USA). Descriptive statistics were computed for all variables. Continuous variables are summarized as means and standard deviations (SDs), while categorical variables are reported as frequencies and percentages.

Given the descriptive nature of the study, the primary analyses focused on characterizing the sample across socio-demographic, clinical, and treatment-related variables. Secondary exploratory analyses examined sex differences and differences between participants fulfilling versus not fulfilling the 2016 ACR criteria.

Sex differences were explored using independent-samples *t*-tests for continuous variables. The assumption of homogeneity of variances was assessed using Levene’s test; when violated, results from the unequal variances (Welch) correction are reported. Given the number of statistical comparisons (*n* = 13), a Bonferroni correction was applied to control for multiple testing, resulting in a corrected significance threshold of *p* < 0.00385 (two-tailed).

Additional exploratory analyses compared participants fulfilling versus not fulfilling the 2016 ACR criteria. Socio-demographic categorical variables were compared using chi-square tests of independence, while age and clinical variables were compared using independent-samples *t*-tests. For clinical comparisons, 16 variables were examined, including WPI and SSS scores, FIQR total and subscale scores, and individual FIQR symptom domains. To control for multiple testing, a Bonferroni correction was applied, resulting in a corrected significance threshold of *p* < 0.0031 (two-tailed). Given the large sample size and the imbalance between groups, effect sizes were calculated to facilitate interpretation. Cohen’s d was computed for pairwise comparisons involving continuous variables and interpreted according to conventional thresholds (0.2 = small, 0.5 = medium, 0.8 = large). For chi-square analyses, Cramer’s V was calculated as a measure of association strength and interpreted according to conventional benchmarks (0.1 = small, 0.3 = medium, 0.5 = large).

### 2.7. Data Quality Control

Data quality control procedures included configuring Google Forms to reduce the likelihood of multiple submissions by disabling the option to submit additional responses after survey completion. In addition, the dataset was screened for duplicate entries by examining identical response patterns across questionnaire items together with timestamp information. No exact duplicate responses were identified. Given the fully anonymous design of the study, no personal identifiers were collected, and all data were analyzed in de-identified form in accordance with a privacy-by-design approach.

## 3. Results

### 3.1. Sample Characteristics

A total of 6216 responses were collected. No exact duplicate responses were identified following data screening procedures. After applying eligibility criteria, 5 participants were excluded because they were under 18 years of age, and 189 were excluded because they self-reported not having received a physician-confirmed diagnosis of fibromyalgia (FM). The final sample consisted of 6022 participants.

The mean age of the sample was 52.32 years (SD = 10.26; range 18–87), based on data available for 5993 participants. The sample was predominantly female (96.7%, *n* = 5822), with males accounting for 3.2% (*n* = 191). Most participants reported living with their current family (70.8%), having completed secondary education (47.2%), and being employed or salaried workers (50.7%). Participants were recruited from all Italian regions, with the highest representation from Sardinia (18.0%), Lombardy (16.2%), and Emilia-Romagna (11.2%). Detailed socio-demographic characteristics are presented in Table 1.

### 3.2. Clinical Characteristics

Regarding clinical characteristics, nearly half of the participants (49.6%) reported experiencing chronic pain for more than 10 years. The most common diagnostic delay was between 1 and 3 years after symptom onset (34.6%). Notably, 50.4% of participants reported having received conflicting medical opinions regarding their diagnosis. In most cases (92.1%), the diagnosis of FM had been made by a rheumatologist.

Based on the 2016 ACR criteria, 79.4% of participants (*n* = 4784) met the criteria for fibromyalgia, while 20.6% of participants (*n* = 1238) did not meet these criteria. Comparisons between participants fulfilling and not fulfilling the 2016 ACR criteria revealed largely comparable socio-demographic profiles, although some statistically significant differences emerged. As expected, participants fulfilling the criteria were slightly younger than those not fulfilling them (51.99 ± 10.23 vs. 53.59 ± 10.27 years; t = 4.90, *p* < 0.001, Cohen’s d = 0.16). Small but statistically significant associations were also observed for education (Cramer’s V = 0.08, *p* < 0.001), occupation (Cramer’s V = 0.07, *p* < 0.001), and time to diagnosis (Cramer’s V = 0.06, *p* < 0.001), whereas no significant differences emerged for sex, housing situation, duration of chronic pain, or the proportion reporting conflicting diagnostic opinions (all *p* > 0.05). Differences in clinical severity were more pronounced. Participants fulfilling the 2016 ACR criteria were more frequently classified in the severe and very severe FIQR categories, whereas those not fulfilling the criteria were more frequently classified in the remission, mild, and moderate categories. This association was statistically significant and of moderate magnitude (χ^2^ = 351.02, *p* < 0.001, Cramer’s V = 0.24; see Supplementary Table S1).

Considering the overall sample, the largest proportion of participants were classified as having severe FM (42.3%) according to FIQR categories. Overall, more than 70% of the sample fell within the moderate-to-high severity range, indicating substantial disease burden. Detailed clinical characteristics are presented in Table 2.

To further characterize these differences, independent-samples *t*-tests were conducted on clinical variables, including WPI, SSS, FIQR scores, and individual symptom domains. Given the number of comparisons performed (*n* = 16), a Bonferroni correction was applied, resulting in an adjusted significance threshold of *p* < 0.0031. Participants fulfilling the 2016 ACR criteria reported significantly higher scores across all clinical measures examined, including WPI, SSS, FIQR total and subscale scores, and all FIQR symptom domains (all *p* < 0.001; see Supplementary Table S2). Effect sizes ranged from small to large, with the largest differences observed for WPI (d = 1.67), FIQR total score (d = 0.64), FIQR functioning (d = 0.63), pain (d = 0.61), SSS (d = 0.59), and FIQR symptoms (d = 0.59).

**Figure 1:**
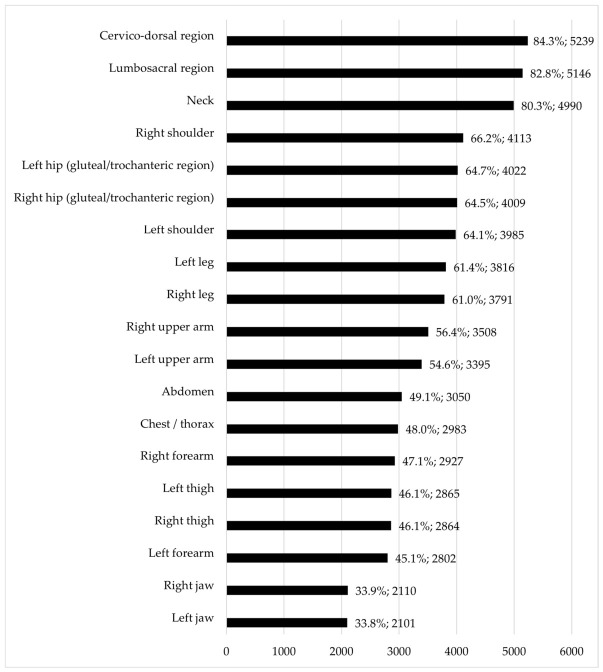
Distribution of Pain Locations According to the Widespread Pain Index (WPI). Participants reported pain across multiple body regions assessed by the WPI. Values represent the number of participants reporting pain in each body region. Multiple responses were allowed; therefore, percentages do not sum to 100%.

Pain was widely distributed across body regions, with the highest frequencies observed in axial areas, including the cervicodorsal region (84.3%), lumbosacral region (82.8%), and neck (80.3%). Peripheral regions were also frequently involved, particularly the shoulders, hips, and legs, each reported by more than 60% of participants. This pattern reflects the widespread and multisite nature of pain in the sample (Figure 1).

Analysis of individual FIQR symptom items showed that the highest scores in the present sample were reported for fatigue (mean = 8.66, SD = 1.66), sleep quality (8.16, SD = 2.18), stiffness (7.96, SD = 1.91), tenderness (7.91, SD = 1.98), and pain (7.84, SD = 1.76). These symptoms were associated with the highest perceived impact on daily life (Table 3).

An additional exploratory analysis investigated whether the pattern of symptom severity across individual FIQR items differed between participants fulfilling and not fulfilling the 2016 ACR criteria. However, no statistically significant differences were observed between groups for any of these symptom items (all *p* > 0.005; see Supplementary Table S2).

### 3.3. Treatments, Lifestyle, and FM Impact on Daily Life

Most participants (81.1%) reported currently using pharmacological treatments for pain management, with a mean perceived effectiveness of 4.50 (SD = 2.29) on a 0–10 scale.

A smaller proportion (26.5%) reported having ever used medical cannabis, with a mean perceived effectiveness of 4.35 (SD = 3.20).

Non-pharmacological treatments were reported by 55.1% of participants. The most frequently reported approaches included physiotherapy (14.3%), gentle exercise (11.0%), and psychotherapy (8.0%), while 15.1% indicated other types of interventions. The overall mean perceived effectiveness of these treatments was 4.90 (SD = 2.29).

Additionally, 60.7% of participants reported having modified their diet following their FM diagnosis. A detailed overview of treatments and perceived effectiveness is provided in Table 4.

Participants reported a substantial impact of FM across multiple domains of daily life. Mean impact scores (0–10 Likert scale) were as follows: family life 7.53 (SD = 2.02), sexual life 7.31 (SD = 2.58), work life 8.07 (SD = 2.03), and social life 7.58 (SD = 2.10).

### 3.4. Sex Differences

Sex differences were examined using independent-samples *t*-tests with Bonferroni correction for multiple comparisons (adjusted significance threshold *p* < 0.00385; Table 5). Overall, most comparisons showed no statistically significant differences between female and male participants after correction for multiple testing. No significant differences emerged for WPI (t = 0.74, *p* = 0.463) or SSS (t = 1.64, *p* = 0.101), indicating similar levels of widespread pain and symptom severity in males and females. Regarding disease impact, females reported significantly higher scores in FIQR functioning (t = 4.79, *p* < 0.001; Cohen’s d = 0.35) and FIQR symptoms (t = 3.23, *p* = 0.001; Cohen’s d = 0.24). The difference in FIQR total score (t = 2.91, *p* = 0.004) did not remain statistically significant after correction for multiple comparisons. No significant difference was observed for the FIQR overall health domain (t = −0.92, *p* = 0.357). With respect to treatment effectiveness, no significant sex differences were found for pharmacological treatments (t = 0.03, *p* = 0.978) or cannabis use (t = −0.42, *p* = 0.675). Females reported slightly higher perceived effectiveness of non-pharmacological treatments, although this difference did not remain significant after correction (t = 2.66, *p* = 0.008). Finally, no significant differences were observed in the perceived impact of fibromyalgia on family (t = −0.90, *p* = 0.367), sexual (t = 1.03, *p* = 0.304), work (t = −1.77, *p* = 0.077), or social life (t = −1.01, *p* = 0.315).

Overall, after correction for multiple comparisons, sex differences were limited to FIQR functioning and FIQR symptom domains, whereas all other differences were not statistically significant. Effect sizes for the significant comparisons were small (Cohen’s d = 0.35 and 0.24, respectively), suggesting limited magnitude of these differences.

## 4. Discussion

The present study characterized a large sample of individuals with FM recruited through an online survey. When these findings are considered alongside published aggregate data from the IFR, several noteworthy similarities and differences emerge.

One of the most notable differences concerns disease severity, which appears to be greater in the online cohort than in the IFR. According to FIQR severity categories, only 6.3% of participants were classified as being in remission or having mild symptoms, compared to 23.6% in the IFR [49]. Conversely, 71.7% of the present sample fell within the severe or very severe categories, whereas this proportion was markedly lower in the IFR (47.4%) [49].

The distribution of pain duration further differentiates the present sample from that of the IFR. In the current study, only 1.4% of participants reported experiencing pain for less than one year, compared to 12.35% in the IFR [23]. Conversely, a large majority of the online sample (71.7%) reported pain lasting six years or more, whereas in the IFR, approximately half of the patients (49.38%) reported a duration of five years or longer [23]. This pattern suggests that the online cohort was characterized by both greater symptom severity and longer pain duration. These findings may be interconnected, as prolonged pain duration may be associated with greater disease severity [50,51].

Additional differences between the two cohorts emerged across clinical measures. Overall, participants in the present study reported higher levels of disease impact across all FIQR domains compared to the IFR [49]. The mean FIQR total score was substantially higher in the online sample (70.89, SD = 17.25) than in the IFR (57.86, SD = 23.37), indicating a greater overall burden of disease. This pattern was consistent across FIQR subdomains. Scores related to functioning (20.29 vs. 16.06), overall health (13.79 vs. 11.06), and symptoms (36.81 vs. 30.74) were all higher in the present sample, suggesting more pronounced impairment in daily activities, poorer perceived health status, and greater symptom severity. A similar trend was observed for symptom-related measures. While WPI scores were broadly comparable between the two samples (11.67 vs. 11.08), indicating a similar extent of pain distribution, SSS scores were notably higher in the online sample (9.61 vs. 7.51). This suggests that, although the distribution of pain is broadly similar, individuals recruited online experience a greater intensity of associated symptoms such as fatigue, cognitive difficulties, and unrefreshing sleep.

A more detailed analysis of individual FIQR symptom items highlights the greater clinical burden observed in the present online sample compared to the IFR [23]. The largest differences were observed for core FM symptoms, including fatigue (8.66 vs. 7.18), sleep disturbances (8.16 vs. 6.87), stiffness (7.96 vs. 6.66), tenderness (7.91 vs. 6.68), and pain (7.84 vs. 6.68). Fatigue and sleep quality, in particular, emerged as the most burdensome symptoms in our online sample, with notably higher scores compared to the IFR. These symptoms are clinically relevant, as they have a pervasive impact on daily functioning and are often reported as among the most disabling aspects of FM [33,52,53]. Interestingly, despite the differences in intensity, the five most burdensome symptoms were the same in both samples—fatigue, sleep disturbances, stiffness, tenderness, and pain—suggesting that the core symptom profile of FM is broadly consistent across populations.

A comparison of socio-demographic characteristics between the present sample and the IFR shows overall similarities between the two populations. The mean age was remarkably close (52.32 years, SD = 10.26 in the present study vs. 51.91 years, SD = 11.5 in the IFR), suggesting a consistent age distribution among individuals with FM regardless of recruitment strategy [49]. Similarly, both samples showed a strong predominance of female participants, reflecting the well-established epidemiology of FM [14,15,16], although the imbalance appears slightly more pronounced in the online sample. In the present study, females accounted for 5822 participants compared to 191 males, while in the IFR, the distribution was 2181 females and 158 males [49]. With regard to education, a relatively high level of educational attainment was observed in both samples. In the present study, 70.4% of participants reported at least a high school diploma or higher education, compared to 65.2% in the IFR [49]. This finding suggests that individuals with higher educational levels may be more likely to participate in both registry-based and survey-based research, possibly due to greater health literacy, awareness of the condition, or engagement with healthcare and research initiatives. Notably, this pattern contrasts with epidemiological data on chronic pain in the Italian general population, where the condition is more prevalent among individuals with lower educational attainment: only 9.4% of people with chronic pain have a university degree or higher, whereas 60.9% have low educational levels [2]. This discrepancy may reflect selection bias, whereby individuals with higher education are more likely to access specialized care (and thus be included in registries) or to engage in online research participation. Additionally, individuals with lower educational attainment might face greater barriers to participation, including limited access to digital tools, lower health literacy, or reduced familiarity with research contexts.

Taken together, these findings suggest that participants recruited through the online survey reported higher levels of symptom severity and pain duration than those described in the IFR, despite broadly similar symptom profiles and socio-demographic characteristics. These similarities provide some contextual support for comparing the two cohorts. Given the descriptive nature of the comparison and the absence of individual-level data allowing adjustment for potential confounders, these findings should be interpreted with caution. Several non-mutually exclusive explanations may account for this pattern. One possible explanation is that individuals experiencing greater symptom burden or longer disease duration may be more likely to seek information, support, or alternative management strategies online and, consequently, more likely to participate in a survey disseminated through these channels [54]. Conversely, individuals with milder symptoms may have been less likely to engage with these recruitment channels. However, because the present study was not designed to directly evaluate recruitment-related selection mechanisms, these interpretations remain speculative. Future studies using individual-level data from both online and registry-based cohorts, or directly comparing different recruitment strategies within the same study design, could employ multivariate and longitudinal approaches to clarify whether the observed differences primarily reflect recruitment-related selection effects, underlying differences in participant characteristics, or a combination of these factors.

Another important finding of the present study is that 79.4% of participants fulfilled the 2016 ACR criteria for FM. These findings suggest that the survey reached a predominantly clinically relevant FM population, while also capturing a subset of individuals who reported a diagnosis of FM but did not meet the 2016 ACR-based classification. Comparisons between participants fulfilling and not fulfilling the 2016 ACR criteria revealed broadly comparable socio-demographic profiles. Although statistically significant differences were observed for age, education, occupation, and time to diagnosis, the magnitude of these associations was small, suggesting limited practical relevance. In contrast, more substantial differences emerged with respect to clinical severity. Participants fulfilling the 2016 ACR criteria reported higher levels of symptom burden across all clinical measures examined and were more frequently classified in the severe and very severe FIQR categories. Although participants not fulfilling the 2016 ACR criteria reported lower symptom severity and disease impact overall, they nevertheless exhibited a substantial clinical burden. Compared with criteria-positive participants, they tended to report less widespread pain, somewhat lower symptom severity, and a lower impact of FM on daily functioning. However, many remained within the moderate or severe FIQR categories, suggesting that the two groups differed primarily in symptom intensity and distribution rather than representing entirely distinct clinical populations. These findings support the view of FM symptomatology as existing along a continuum of severity [55,56,57], with diagnostic thresholds serving as operational cut-offs within a graded distribution of symptoms. In line with these findings, Srinivasan et al. [58] reported limited agreement between physician-based and criteria-based FM diagnosis. In their study, only about one-third of patients with a physician diagnosis of FM met the 2016 ACR criteria, while only one-third of criteria-positive patients had a corresponding physician diagnosis. The mean PSD scores were 12.4 and 18.4 in the physician-diagnosed and criteria-based groups, respectively, indicating meaningful differences in symptom burden between diagnostic categories. A similar pattern emerged in the present study, where participants fulfilling the 2016 ACR criteria reported substantially higher symptom severity than those not fulfilling the criteria across multiple clinical domains. These findings highlight the value of standardized instruments and criteria-based classification in FM research. The use of the 2016 ACR criteria facilitates comparability across studies and enables a more consistent characterization of patient populations. From a clinical perspective, the findings also support the potential utility of structured assessment tools such as the WPI and SSS for systematically evaluating symptom burden and complementing clinical judgement. Future research should further investigate whether the routine use of these instruments may improve patient stratification and support more individualized management strategies.

Sex-stratified analyses in the present online sample revealed largely comparable clinical profiles between female and male participants. After correction for multiple comparisons, statistically significant differences were limited to FIQR functioning and FIQR symptom domains, with females reporting slightly higher levels of impairment and symptom severity. However, effect sizes were generally small, and differences were not consistently observed across clinical domains, suggesting limited clinical relevance of sex-related variations in this cohort. When considered alongside published IFR aggregate data, our online cohort appears to show a broadly similar pattern of sex-related differences, although the magnitude of differences between females and males appears less pronounced in several domains compared to registry-based data. Specifically, IFR data suggest a somewhat wider separation between sexes in FIQR scores than observed in the present sample. However, these comparisons remain descriptive, as IFR data were available only at the aggregate level and could not be adjusted for potential confounding variables. Therefore, no formal inference can be made regarding differences across recruitment modalities. Future studies using individual-level data and more balanced sex distributions are needed to better clarify the extent and clinical relevance of sex-related differences in FM across different recruitment settings.

Another notable finding is that 50.4% of participants reported receiving conflicting medical opinions regarding their diagnosis. This high rate of diagnostic uncertainty is consistent with previous literature and points to the ongoing challenges in recognizing and managing FM [20,59]. It suggests that many patients face fragmented care, which may contribute to delays in diagnosis, patient frustration, and potentially greater symptom burden [11,60,61]. These findings emphasize the importance of improving diagnostic pathways and promoting more consistent and standardized approaches to FM assessment across healthcare settings.

Another finding deserving consideration is the relatively low perceived effectiveness reported across treatment modalities. Mean effectiveness ratings were below the midpoint of the scale for pharmacological treatments, medical cannabis, and non-pharmacological interventions, suggesting that many participants continue to experience a substantial symptom burden despite ongoing treatment. This observation is consistent with the well-recognized challenges of managing FM, a condition for which currently available treatments often provide only partial symptom relief [6,60,62]. At the same time, these findings should be interpreted cautiously. Treatment effectiveness was assessed solely through patient self-report and did not account for treatment type, dosage, duration, adherence, treatment combinations, or baseline disease severity. Consequently, the reported scores should be interpreted as indicators of perceived benefit rather than objective measures of treatment efficacy. This consideration is particularly relevant for medical cannabis, for which no information was available regarding formulation, THC/CBD composition, dosage, duration of use, or prescribing indication. Future studies should examine treatment-related variables in greater detail to better understand the factors associated with perceived treatment effectiveness in FM.

### Strength and Limitations

A key strength of the present study lies in the characterization of one of the largest Italian online cohorts of individuals with FM described to date. The large sample size enabled a detailed description of socio-demographic, clinical, and treatment-related characteristics across a broad range of variables. Furthermore, the inclusion of the components required to operationalize the 2016 ACR criteria allowed the application of a standardized and reproducible criteria-based classification procedure, strengthening the clinical characterization of the sample. In addition, the application of the 2016 ACR criteria enabled the characterization of both criteria-positive and criteria-negative participants, providing additional insight into the clinical heterogeneity of individuals self-identifying as having FM. Although recruitment through patient organizations, online communities, and social media networks may introduce selection effects, these strategies may provide access to segments of the FM population that could be underrepresented in clinical registries and healthcare-based samples. Importantly, despite reporting substantially greater symptom burden than participants in the IFR, the overall symptom profile and demographic characteristics of the online cohort remained broadly similar. From a methodological perspective, these findings support the view that online surveys and clinical registries may provide complementary perspectives on the same underlying condition, each contributing unique information regarding different segments of the FM population.

Despite its strengths, the present study has several limitations that should be considered when interpreting the results. First, the comparison with the IFR was based on published aggregate data, as individual-level IFR data were not available. Consequently, multivariate analyses adjusting for potential confounders could not be performed, and comparisons between the two samples should be considered descriptive and exploratory rather than inferential. Second, the cross-sectional design of the study precludes any conclusions regarding causality or changes in symptom severity over time. Longitudinal studies are needed to clarify temporal relationships between disease duration, symptom burden, and clinical outcomes. Third, while online recruitment allowed access to a large and heterogeneous sample of individuals with FM, the resulting study population should not be considered representative of the broader Italian FM population. Recruitment relied heavily on patient organizations, social media platforms, online communities, and disease-specific networks, which may have preferentially reached individuals who were more engaged with their condition or more motivated to participate in research. Consequently, the symptom burden observed in the present sample should not be interpreted as an epidemiological estimate of disease severity among all individuals with FM. The regional distribution of participants also suggests that our survey may not be fully representative of the Italian FM population. In particular, Sardinia accounted for a disproportionately large share of respondents relative to its population size. This pattern may reflect the strong involvement of local CFU-Italia representatives in survey dissemination, the historically high participation of Sardinian patients in fibromyalgia-related initiatives, or greater regional awareness of the condition.

Additionally, several variables, including pain duration and the time interval between symptom onset and FM diagnosis, were based on retrospective self-report. Given that many participants reported symptom histories extending over several years, and frequently more than a decade, some degree of recall bias may have affected the accuracy of these estimates. FM diagnosis was also based on participant self-report and could not be independently verified. Although respondents indicating that they had not received a physician diagnosis were excluded and information regarding the diagnosing specialist was collected, a small proportion of participants may have reported provisional, hypothesized, or inaccurately recalled diagnoses. Nevertheless, the majority of participants fulfilled the 2016 ACR criteria, supporting the overall clinical validity of the sample. Furthermore, because data on survey views and partial completions were not available, a survey completion rate could not be calculated, and the potential impact of non-response bias could not be formally assessed. Finally, the marked imbalance between female and male participants limits the precision and stability of sex-stratified analyses, which should therefore be interpreted with caution.

## 5. Conclusions

In conclusion, the present study provides a comprehensive characterization of one of the largest Italian online cohorts of individuals with FM described to date. Participants reported substantial symptom burden, long pain duration, and marked impairment across multiple domains of functioning and well-being. The application of the 2016 ACR criteria also enabled a standardized criteria-based classification of participants and provided additional insight into the clinical heterogeneity of individuals self-identifying as having FM. Compared with published data from the IFR, our online cohort exhibited greater symptom severity and disease impact while maintaining a broadly similar symptom profile and socio-demographic composition. However, the extent to which the observed differences reflect recruitment-related selection effects, underlying differences in participant characteristics, or other factors cannot be determined from the present data. Overall, the findings support the value of standardized assessment tools and criteria-based classification procedures in FM research and suggest that integrating evidence from different recruitment settings may contribute to a more comprehensive understanding of the heterogeneity of fibromyalgia populations.

## Figures and Tables

**Table 1:** Socio-demographic characteristics of the study sample (N = 6022).

Variable	N (%)	Mean (SD)
**Age**	—	52.32 (10.26)
**Sex**		
Female	5822 (96.7)	—
Male	191 (3.2)	—
Other	9 (0.1)	—
**Housing situation**		
Living with others	221 (3.7)	—
Living with current family	4264 (70.8)	—
Living with family of origin	584 (9.7)	—
Living alone	953 (15.8)	—
**Education**		
Primary/lower secondary/vocational	1780 (29.6)	—
High school diploma	2842 (47.2)	—
University degree	1014 (16.8)	—
Postgraduate degree	386 (6.4)	—
**Occupation**		
Housewife/househusband	875 (14.5)	—
Unemployed	886 (14.7)	—
Employee	3054 (50.7)	—
Self-employed	484 (8.0)	—
Retired	663 (11.0)	—
Student	60 (1.0)	—
**Region**		
Sardinia	1081 (18.0)	—
Lombardy	976 (16.2)	—
Emilia-Romagna	676 (11.2)	—
Tuscany	383 (6.4)	—
Lazio	375 (6.2)	—
Veneto	345 (5.7)	—
Piedmont	335 (5.6)	—
Puglia	268 (4.5)	—
Campania	267 (4.4)	—
Sicily	237 (3.9)	—
Friuli Venezia Giulia	204 (3.4)	—
Liguria	195 (3.2)	—
Marche	165 (2.7)	—
Calabria	156 (2.6)	—
Umbria	120 (2.0)	—
Abruzzo	75 (1.2)	—
Basilicata	73 (1.2)	—
Trentino-Alto Adige	65 (1.1)	—
Valle d’Aosta	15 (0.2)	—
Molise	11 (0.2)	—

Bold text indicates the main variable categories and is used for readability purposes only.

**Table 2:** Clinical characteristics of the study sample (N = 6022).

Variable	N (%)	Mean (SD)
**Duration** **of chronic pain**		
<1 year	86 (1.4)	—
1–3 years	748 (12.4)	—
4–5 years	872 (14.5)	—
6–10 years	1329 (22.1)	—
>10 years	2987 (49.6)	—
**Time to diagnosis (time lag)**		
<1 year	988 (16.4)	—
1–3 years	2085 (34.6)	—
4–5 years	983 (16.3)	—
6–10 years	891 (14.8)	—
>10 years	1065 (17.7)	—
**Conflicting diagnostic opinions**		
No	2986 (49.6)	—
Yes	3036 (50.4)	—
**Diagnosing physician**		
Rheumatologist	5549 (92.1)	—
Neurologist	183 (3.0)	—
Pain specialist	121 (2.0)	—
Other	127 (2.1)	—
Orthopedic specialist	42 (0.7)	—
**Clinical scores**		
WPI	—	11.67 (4.83)
SSS	—	9.61 (1.71)
FIQR total	—	70.89 (17.25)
FIQR Functioning	—	20.29 (6.21)
FIQR Overall health	—	13.79 (4.99)
FIQR Symptoms	—	36.81 (7.80)
**FIQR severity categories**		
Remission	69 (1.1)	—
Mild	316 (5.2)	—
Moderate	1323 (22.0)	—
Severe	2546 (42.3)	—
Very severe	1768 (29.4)	—

Note. WPI = Widespread Pain Index; SSS = Symptom Severity Scale; FIQR = Fibromyalgia Impact Questionnaire—Revised. Bold text indicates the main variable categories and is used for readability purposes only.

**Table 3:** Descriptive statistics for individual FIQR symptom items (N = 6022).

Symptom	Mean (SD)
Pain	7.84 (1.76)
Fatigue	8.66 (1.66)
Stiffness	7.96 (1.91)
Sleep quality	8.16 (2.18)
Depression	5.88 (2.85)
Memory problems	6.78 (2.41)
Anxiety	6.49 (2.77)
Tenderness	7.91 (1.98)
Balance problems	6.32 (2.62)
Environmental sensitivity	7.60 (2.26)

**Table 4:** Pharmacological and non-pharmacological treatments and their perceived effectiveness.

Variable	N (%)	Mean (SD)
**Pharmacological** **treatment use**		
No	1138 (18.9)	—
Yes	4884 (81.1)	—
**Pharmacological treatment effectiveness**	—	4.50 (2.29)
**Cannabis use**		
No	4426 (73.5)	—
Yes	1596 (26.5)	—
**Cannabis effectiveness**	—	4.35 (3.20)
**Non-pharmacological treatments**		
No	2703 (44.9)	—
Yes	3319 (55.1)	—
**Type of non-pharmacological treatments**		
Physiotherapy	863 (14.3)	—
Gentle exercise	661 (11.0)	—
Psychotherapy	481 (8.0)	—
Other	909 (15.1)	—
Yoga	253 (4.2)	—
Acupuncture	232 (3.9)	—
Spa treatments	203 (3.4)	—
Meditation	195 (3.2)	—
Phytotherapy	126 (2.1)	—
Tai chi	39 (0.6)	—
**Non-pharmacological treatment effectiveness**	—	4.90 (2.29)

Bold text indicates the main variable categories and is used for readability purposes only.

**Table 5:** Sex differences in clinical characteristics, perceived treatment effectiveness, and disease impact.

Variable	Females Mean (SD)	Males Mean (SD)	t	*p*	Cohen’s d
WPI	11.68 (4.81)	11.40 (5.19)	0.74	0.463	0.06
SSS	9.62 (1.71)	9.41 (1.72)	1.64	0.101	0.12
FIQR Functioning	20.36 (6.19)	18.18 (6.45)	4.79	<0.001	0.35
FIQR Symptoms	36.87 (7.80)	35.02 (7.81)	3.23	0.001	0.24
FIQR Health	13.78 (4.99)	14.12 (4.76)	−0.92	0.357	−0.07
FIQR Total	71.01 (17.25)	67.32 (17.02)	2.91	0.004	0.21
Pharmacological treatment effectiveness	4.50 (2.28)	4.50 (2.37)	0.03	0.978	0.00
Cannabis effectiveness	4.35 (3.21)	4.51 (3.05)	−0.42	0.675	−0.05
Non-pharmacological treatment effectiveness	4.92 (2.29)	4.40 (2.28)	2.66	0.008	0.23
Family impact	7.53 (2.03)	7.66 (1.85)	−0.90	0.367	−0.07
Sexual life impact	7.32 (2.59)	7.12 (2.38)	1.03	0.304	0.08
Work impact	8.06 (2.04)	8.33 (1.77)	−1.77	0.077	−0.14
Social life impact	7.58 (2.10)	7.73 (1.96)	−1.01	0.315	−0.07

Note. WPI = Widespread Pain Index; SSS = Symptom Severity Scale; FIQR = Fibromyalgia Impact Questionnaire—Revised. Cohen’s d values indicate effect size (0.2 = small, 0.5 = medium, 0.8 = large).

## Data Availability

The data presented in this study are available upon request from the corresponding author.

## Supplementary Materials

The following supporting information can be downloaded at https://www.mdpi.com/article/10.3390/medicina62071319/s1, Table S1: Socio-demographic and clinical characteristics of participants fulfilling versus not fulfilling the 2016 ACR criteria; Table S2: Comparison of clinical severity measures between participants fulfilling versus not fulfilling the 2016 ACR criteria.

## Abbreviations

The following abbreviations are used in this manuscript:

ACR	American College of Rheumatology
FIQR	Fibromyalgia Impact Questionnaire Revised
FM	Fibromyalgia
ICD	International Classification of Diseases
IFR	Italian Fibromyalgia Registry
SSS	Symptom Severity Scale
WPI	Widespread Pain Index
