# Supplementary material for: Self-Reported Symptom Burden and Clinical Characteristics in Fibromyalgia: Evidence from a Large Online Survey in Italy
Source: Medicina (Kaunas). 2026 Jul 8;62(7):1319. doi: 10.3390/medicina62071319 (PMC13414456; doi:10.3390/medicina62071319)
Supplement: Supplementary file 1 [file medicina-62-01319-s001.zip › Table S2.pdf]

Table S2. Comparison of Clinical Severity Measures Between Participants Fulfilling versus Not Fulfilling the 2016 ACR Criteria.

Variable	Participants Not Fulfilling 2016 ACR Criteria (N=1238)	Participants Fulfilling 2016 ACR Criteria (N=4784)	t	p	Cohen's d
	**Mean (SD)**	**Mean (SD)**			
**SSS**	8.84 (1.94)	9.81 (1.59)	-16.315	<0.001	-0.59
**WPI**	6.22 (2.70)	13.08 (4.21)	-69.920	<0.001	-1.67
**Pain**	7.05 (2.07)	8.05 (1.60)	-15.839	<0.001	-0.61
**Fatigue**	8.00 (2.08)	8.83 (1.48)	-13.120	<0.001	-0.54
**Stiffness**	7.26 (2.19)	8.15 (1.79)	-13.178	<0.001	-0.49
**Sleep quality**	7.52 (2.47)	8.33 (2.06)	-10.539	<0.001	-0.38
**Depression**	5.28 (2.94)	6.04 (2.81)	-8.232	<0.001	-0.26
**Memory problems**	6.20 (2.60)	6.93 (2.33)	-9.030	<0.001	-0.31
**Anxiety**	5.96 (2.91)	6.5630 (2.71)	-7.313	<0.001	-0.21
**Tenderness**	7.04 (2.31)	8.13 (1.82)	-15.390	<0.001	-0.58
**Balance problems**	5.50 (2.87)	6.53 (2.51)	-11.508	<0.001	-0.41
**Environmental sensitivity**	6.88 (2.59)	7.79 (2.13)	-11.394	<0.001	-0.42
**FIQR-Functioning**	17.36 (6.82)	21.05 (5.80)	-17.450	<0.001	-0.63
**FIQR Overall health**	11.89 (5.51)	14.29 (4.72)	-14.062	<0.001	-0.50
**FIQR Symptoms**	33.35 (8.88)	37.70 (7.23)	-15.949	<0.001	-0.59
**FIQR-Total score**	62.59 (19.37)	73.04 (15.97)	-17.498	<0.001	-0.64
