# Supplementary material for: Self-Reported Symptom Burden and Clinical Characteristics in Fibromyalgia: Evidence from a Large Online Survey in Italy
Source: Medicina (Kaunas). 2026 Jul 8;62(7):1319. doi: 10.3390/medicina62071319 (PMC13414456; doi:10.3390/medicina62071319)
Supplement: Supplementary file 1 [file medicina-62-01319-s001.zip › Table S1.pdf]

Table S1. Socio-Demographic and Clinical Characteristics of Participants Fulfilling versus Not Fulfilling the 2016 ACR Criteria.

Variable	Participants Not Fulfilling 2016 ACR Criteria (N=1238)		Participants Fulfilling 2016 ACR Criteria (N=4784)		p
	**Mean (SD)**	**N (%)**	**Mean (SD)**	**N (%)**	
**Age**	53.59 (10.27)		51.99 (10.23)		<0.001
**Sex**					0.623
Female		1194 (96.4%)		4628 (96.7%)	
Male		43 (3.5%)		148 (3.1%)	
Other		1 (0.1%)		8 (0.2%)	
**Housing situation**					0.165
Living with others		50 (4%)		171 (3.6%)	
Living with current family		849 (68.6%)		3415 (71.4%)	
Living with family of origin		120 (9.7%)		464 (9.7%)	
Living alone		219 (17.7%)		734 (15.3%)	
**Education**					<0.001
Primary/lower secondary/vocational		285 (23%)		1495 (31.3%)	
High school diploma		613 (49.5%)		2229 (46.6%)	
University degree		236 (19.1%)		778 (16.3%)	
Postgraduate degree		104 (8.4%)		282 (5.9%)	
**Occupation**					<0.001
Housewife/househusband		191 (15.4%)		684 (14.3%)	
Unemployed		131 (10.6%)		755 (15.8%)	
Employee		639 (51.6%)		2415 (50.5%)	
Self-employed		106 (8.6%)		378 (7.9%)	
Retired		163 (13.2%)		500 (10.5%)	
Student		8 (0.6%)		52 (1.1%)	
**Duration of chronic pain**					0.276
< 1 year		23 (1.9%)		63 (1.3%)	
1-3 years		145 (14.2%)		603 (12.6%)	
4-5 years		176 (14.2%)		696 (14.5%)	
6-10 years		294 (23.7%)		1035 (21.6%)	
> 10 years		600 (48.5%)		2387 (49.9%)	
**Time to diagnosis (time lag)**					<0.001
< 1 year		236 (19.1%)		752 (15.7%)	
1–3 years		450 (36.3%)		1635 (34.2%)	
4–5 years		160 (12.9%)		823 (17.2%)	
6–10 years		180 (14.5%)		711 (14.9%)	
> 10 years		208 (16.8%)		857 (17.9%)	
**Conflicting diagnostic opinions**					0.158
No		636 (51.4%)		2350 (49.1%)	
Yes		602 (48.6%)		2434 (50.9%)	
**FIQR severity categories**					<0.001
Remission		46 (3.7%)		23 (0.5%)	
Mild		138 (11.1%)		178 (3.7%)	
Moderate		388 (31.3%)		935 (19.5%)	
Severe		460 (37.2%)		2086 (43.6%)	
Very severe		206 (16.6%)		1562 (32.7%)	
